# Unconjugated Multi-Epitope Peptides Adjuvanted with ALFQ Induce Durable and Broadly Reactive Antibodies to Human and Avian Influenza Viruses

**DOI:** 10.3390/vaccines11091468

**Published:** 2023-09-08

**Authors:** Nimisha Rikhi, Clara J. Sei, Mangala Rao, Richard F. Schuman, Kellie A. Kroscher, Gary R. Matyas, Kevin Muema, Camille Lange, Aba Assiaw-Dufu, Elizabeth Hussin, Ousman Jobe, Carl R. Alving, Gerald W. Fischer

**Affiliations:** 1 Longhorn Vaccines and Diagnostics, Gaithersburg, MD 20878, USA; nr@lhnvd.com (N.R.); kellie@lhnvd.com (K.A.K.); kevin@lhnvd.com (K.M.); aba@lhnvd.com (A.A.-D.); gwf@lhnvd.com (G.W.F.); 2 U.S. Military HIV Research Program, Walter Reed Army Institute of Research, Silver Spring, MD 20910, USA; mrao@hivresearch.org (M.R.); gmatyas@hivresearch.org (G.R.M.); clange@hivresearch.org (C.L.); elizabethhussin17@gmail.com (E.H.); ojobe@hivresearch.org (O.J.); calving@hivresearch.org (C.R.A.); 3 Antibody and Immunoassay Consultants, Rockville, MD 20850, USA; rfs@aicbiotech.com; 4 Henry M Jackson Foundation for the Advancement of Military Medicine, Bethesda, MD 20817, USA

**Keywords:** peptides, influenza, ALFQ, liposomes, QS21 (also known as QS-21), universal vaccine, durable, immune responses, intramuscular, intradermal, broadly reactive antibodies, neutralizing antibodies

## Abstract

An unconjugated composite peptide vaccine targeting multiple conserved influenza epitopes from hemagglutinin, neuraminidase, and matrix protein and formulated with a safe and highly potent adjuvant, Army Liposome formulation (ALFQ), generated broad and durable immune responses in outbred mice. The antibodies recognized specific epitopes in influenza peptides and several human, avian, and swine influenza viruses. Comparable antibody responses to influenza viruses were observed with intramuscular and intradermal routes of vaccine administration. The peptide vaccine induced cross-reactive antibodies that recognized influenza virus subtypes A/H1N1, A/H3N2, A/H5N1, B/Victoria, and B/Yamagata. In addition, immune sera neutralized seasonal and pandemic influenza strains (Group 1 and Group 2). This composite multi-epitope peptide vaccine, formulated with ALFQ and administered via intramuscular and intradermal routes, provides a high-performance supra-seasonal vaccine that would be cost-effective and easily scalable, thus moving us closer to a viable strategy for a universal influenza vaccine and pandemic preparedness.

## 1. Introduction

The current seasonal influenza vaccines are made each year to match the strains expected to be in circulation in the upcoming flu season. This is necessary to circumvent the evolutionary nature of influenza viruses, which undergo routine antigenic drift and shift. The traditional method of influenza vaccine production using embryonated chicken eggs often leads to mismatch between the egg-adapted virus strains and the circulating strains, thereby reducing the protection conferred by the vaccine. Additionally, the process of vaccine production in eggs is time-consuming and does not accommodate pandemic response [1,2,3,4]. Therefore, the call for improved vaccine strategies remains a public health priority. Current vaccines that are in developmental or clinical trial stages include virus-like particle (VLP)-based vaccines, nanoparticle-based vaccines, viral vector-based vaccines (e.g., adenovirus vector), and DNA or mRNA-based vaccines [4,5,6,7,8,9,10,11,12,13]. Synthetic, multi-epitope peptide vaccines that offer rapid and cost-effective advantages in scaling-up for clinical trials are rapidly gaining prominence as a universal influenza vaccine strategy [5]. A potential disadvantage of small peptide vaccines is their lack of immunogenicity, which can be circumvented via conjugation with immunogenic carrier molecules such as the mutated diphtheria toxin CRM197, or through formulation with safe and highly potent adjuvants such as the liposome-based ALFQ adjuvant, as well as the incorporation of universal T-cell epitopes in the peptide sequence for intrinsic immune-stimulation [14,15,16,17,18].

Seasonal and pandemic influenza both cause severe disease. The spread of highly pathogenic avian influenza virus (A/H5N1) in poultry and its transmission to humans has posed a threat of another pandemic if the outbreaks in poultry are not controlled [19]. Additionally, in humans and pigs, influenza B strains (B/Victoria and B/Yamagata) also co-circulate with influenza A in seasonal outbreaks and have been involved in influenza epidemics [20,21,22]. We have already witnessed a previous zoonotic influenza pandemic in 2009, with the H1N1 swine flu triple-reassortant strain [23,24,25]. In 2020, gene assortments from the 2009 swine flu H1N1 pandemic strain demonstrated high affinity binding to human-like Saα2, 6Gal receptors, which is a prerequisite for human infection [26]. Therefore, a universal influenza vaccine with cross-neutralizing antibodies against mammalian, swine and avian influenza strains would greatly help in preventing a future pandemic via zoonotic influenza transmission.

Previously, we demonstrated that 1 µg of an unconjugated peptide vaccine consisting of highly conserved composite HA, NA, Matrix (M1/M2/M2e) epitopes and a tetanus toxoid T-cell epitope formulated with Army Liposome Formulation with QS-21 (ALFQ) adjuvant elicited robust immune responses in mice when administered via an intramuscular route [14]. Here, we demonstrate that this influenza vaccine generated durable and broadly reactive antibodies in outbred mice at a low dose (1 µg) and a high dose (20 µg) administered intramuscularly and intradermally. In addition, the antisera recognized several subtypes of human, swine and avian influenza A and influenza B viruses and exhibited neutralizing activity against Group 1 and Group 2 influenza strains.

In this study, we corroborate our previous observation of robust immunogenicity with the low-dose (1 µg) vaccine and further extend our knowledge concerning the durability of immune responses generated via multiple routes of administration and cross-reacting with multiple subtypes of influenza viruses, including the highly pathogenic avian influenza A/H5N1 strains and influenza B strains.

## 2. Materials and Methods

### 2.1. Cell Culture

Madin-Darby canine kidney (MDCK) cells (ATCC, Manassas, VA, USA) were maintained in Eagle’s Minimal Essential Medium (EMEM) (Lonza, Basel, Switzerland), and MDCK-ATL cells (Influenza Reagents Resource (IRR) Manassas, VA, USA) in Dulbecco’s Modified Eagle Medium (DMEM) (Fisher Scientific, Pittsburg, PA, USA), with each medium containing 5% fetal bovine serum (FBS) (Cytiva/Hyclone, Marlborough, MA, USA), 2 mM L-glutamine and 1mM sodium pyruvate (Sigma-Aldrich, St. Louis, MO, USA), 1X non-essential amino acids (NEAA) (Corning, Corning, NY, USA), 2.5 µg/mL amphotericin B (Lonza, Walkersville, MD, USA or, Cytiva/Hyclone, Marlborough, MA, USA) and 50 µg/mL gentamycin (Corning, Corning, NY, USA).

### 2.2. Influenza Viruses

Several influenza viruses were obtained from the Influenza Reagents Resource (IRR) (Manassas, VA, USA), established by the Centers for Disease Control and Prevention (CDC), USA. Influenza virus strains used to determine vaccine-induced cross-reactive immunity and neutralization were A/Cal/07/09pdm09 (H1N1), A/Cal/04/09pdm09 (H1N1), A/Mich/45/15pdm09 (H1N1), A/Wisc/505/18pdm09 (H1N1), A/HK/4801/14 (H3N2), A/Vict/361/11 (H3N2), A/Texas/50/12 (H3N2), A/Ind/NIV/06 (H5N1), A/Egypt/321/07 (H5N1), A/Anhui/01/05 (H5N1), A/Vietnam/1203/04 (H5N1), A/Hubei/01/10 (H5N1), A/chicken/Vietnam/16/08 (H5N1), B/NHamp/01/21 (BV), B/OK/10/18 (BY) and B/Bangld/5972/07 (BY) (for complete virus ID and clade, refer to Supplemental Table S1). All viruses were propagated by the infection of MDCK monolayers for 3 days at 37 °C and 5% CO_2_. Virus concentrations were determined by the standard tissue culture infectious dose (TCID_50_) assay on MDCK cells [27] and aliquots were stored at −80 °C.

### 2.3. Influenza Peptides

The composite influenza peptides (referred to in this article as LHNVD-105) was used for mice immunization studies. LHNVD-105 has previously been described in detail [14]. It comprised two composite influenza peptides, HA + NA (Flu Pep11) and Matrix (M1/M2/M2e) + T-cell epitope (Flu Pep5906), adjuvanted with ALFQ. HA + NA (Flu Pep11) comprised two HA epitopes (GNLFIAP and WGVIHHP) and an NA epitope (HYEECSCY), and Matrix (M1/M2/M2e) + T-cell epitope (Flu Pep5906) comprised M1/M2/M2e epitope (SLLTEVETPIRNEWGLLTEVETPIR) and a tetanus T-cell epitope (QYIKANSKFIGITE) [14]. The individual and composite peptides were synthesized under a contract with Atlantic Peptides, Lewisburg, PA, USA and used in immunoassays to evaluate binding activities of anti-influenza antibodies. 

### 2.4. ALFQ Formulation with Composite Influenza Peptides

ALFQ was manufactured as described previously [14,28,29]. Briefly, ALFQ consisted of DMPC, DMPG, cholesterol, MPLA and QS21. The bulk phospholipids (DMPC + DMPG) were present as 45.8 mM relative to Sorensen’s phosphate-buffered saline pH 6.2 (SPBS). The molar ratios of DMPC:DMPG:Cholesterol:MPLA:QS21 were 9:1:12.2:0.114:0.044.

Lyophilized Flu Pep11 and Flu Pep5906 were individually reconstituted in Dulbecco’s phosphate-buffered saline pH 7.4 (DPBS) to achieve a concentration of 1.6 mg/mL per peptide. Two different formulations were created: 1 µg dose (low-dose) and 20 µg dose (high-dose).

Low-dose or high-dose LHNVD-105/ALFQ vaccines were formulated by adding 4.375 µL or 87.5 mL, respectively, of each reconstituted peptide and 175 µL of ALFQ (1.145 mmol of ALFQ total phospholipids/dose) to a sterile vial. To equalize the volume, 166.25 µL DPBS was added to the low-dose vaccine. Vaccine formulations were vortexed at a slow speed for 30 s, and then put on a roller for 15 min to complete mixing. The mixed vaccine formulations were then stored at 4 °C for 16 h prior to immunization. Each mouse was injected with 50 µL of low- or high-dose vaccine. The same formulations were used for intramuscular and intradermal administration routes.

### 2.5. Murine Immunizations using ALFQ Formulated Composite Peptides (LHNVD-105)

Animal studies were carried out in accordance with the recommendations in the Guide for the Care and Use of Laboratory Animals of the National Institutes of Health. All animal procedures were approved by the Institutional Animal Care and Use Committee (Sigmovir Biosystems Inc., Rockville, MD, USA, IACUC Protocol #71). Female Institute of Cancer Research (ICR-CD-1^®^) mice, 3–4 weeks of age, were obtained from Envigo (Indianapolis, IN, USA) for use in this study, as described previously [14]. The composite unconjugated influenza peptides (Flu Pep11 + Flu Pep5906, in combination, referred to as LHNVD-105), formulated with ALFQ adjuvant, were injected either intramuscularly or intradermally at doses of 1 or 20 µg/mouse (n = 5mice/dose/group). Mice were immunized on day 0 and booster immunizations were given on days 21 and 35. Sera from submandibular bleeds were obtained on day -1 (pre-immunization), and days 21, 28, 35, 42, 49, 56, 63, 99, 154 and 200. Approximately 100–150 µL of blood was collected at each bleed and processed for serological testing.

### 2.6. Antisera ELISA: Detection of Antibodies That Bind to Influenza Peptides and Viruses

Serum anti-influenza levels were evaluated using peptides or live/inactivated influenza viruses, using the procedure previously described [14]. Briefly, 96-well NUNC MaxiSorp ELISA plates (Fisher Scientific, Pittsburg, PA, USA) were coated with peptides (1 µg/mL) or influenza viruses (10^5^ TCID_50_ per mL) in PBS (100 µL per well) for 18–24 h at room temperature (RT) and 2–8 °C, respectively. Serial dilutions of serum samples starting at 1:100, with five-fold dilutions (1:100, 1:500, 1:2500, 1:12,500, etc.), were added to antigen-coated plates blocked with 3% normal goat serum (NGS, Southern Biotech, Birmingham, AL, USA) and detected with Horse Radish Peroxidase (HRP)-conjugated isotype-specific goat anti-mouse IgG (Southern Biotech, Birmingham, AL, USA). TMB Substrate Solution (Fisher Scientific, Pittsburg, PA, USA) was added prior to being quenched with TMB STOP solution (Fisher Scientific, Pittsburg, PA, USA). The absorbance values (450 nm) of each well were obtained using a SpectraMax Plus Microplate Reader (Molecular Devices, Sunnyvale, CA, USA). Data were expressed as endpoint titers defined as the reciprocal of highest dilution that yielded an absorbance value greater than or equal to twice that of the pooled pre-immune serum. Due to a minimum starting serum dilution of 1:100, titers below 100 were reported as nil.

### 2.7. Microneutralization Assay (MNA) for Determination of Serum Neutralizing Antibodies

MDCK cells were seeded for 3–4 days in 96-well plates (Corning, Tewksbury, MA, USA). Pooled anti-influenza sera samples were diluted 1:640 in plain EMEM medium, added to Costar^®^ Spin-X^®^ centrifuge tube filters (Corning, New York, NY, USA), heat-inactivated at 56 °C for 30 min and centrifuged at 8000 rpm (5900× *g*) for 2 min. Sera were subsequently serially diluted two-fold from the starting dilution of 1:640 in TPCK-trypsin medium in a 96-well plate. Then, 120 µL per well of influenza A viruses (H3N2 or H1N1) was added to each well containing 120 µL of diluted antisera and incubated for 2 h at 37 °C/5% CO_2_. This addition of an equal volume of virus into the anti-influenza sera samples further increased the antisera dilution two-fold to a starting dilution of 1:1280 and a final dilution of 1:81,920. Confluent MDCK cell monolayers in 96-well plates were washed with PBS (Fisher Scientific, Pittsburg, PA, USA) using an ELx405 Automated Plate Washer (BioTek, Winooski, VT, USA) 10 min before the next step. After 2 h incubation, 100 µL/well of each dilution of antisera-virus mixture was transferred to quadruplicate wells of pre-washed MDCK cell monolayers. Wells containing virus dilutions only and MDCK cells only were included in each plate as positive and negative controls for infectivity, respectively. The assay plates were incubated at 37 °C/5% CO_2_ for 4 days. After incubation, the plates were stained with Crystal Violet and scored by recording the cytopathic effect (CPE) of the virus on the MDCK cell monolayer. Clear wells representing an absence of MDCK cells were scored as positive CPE and indicated an absence of neutralization of viral replication by the antisera tested. Wells with a blue coloration representing the presence of MDCK cells were scored as negative CPE and indicated neutralization of viral replication by the antisera tested. Neutralizing titers were reported as reciprocal of the highest dilution of serum that showed neutralization of influenza virus in three out of four wells with the MDCK cell monolayer.

### 2.8. Phylogenetic Reconstructions of Influenza Strains

Publicly available nucleotide sequences of H1N1, H3N2, H5Nx and B/Victoria influenza strains (210 sequences), commercial seasonal vaccines and the EpiFlu^TM^ global phylodynamic reconstructions were retrieved from the Global Initiative on Sharing All Influenza Data (GISAID) (accessed on 23 May 2023) [30]. All sequences were visualized in AliView (www.ormbunkar.se/aliview) (downloaded on 24 November 2021) and aligned with Multiple Alignment using Fast Fourier Transform (MAFTT) [31]. Neighbor-joining trees based on pairwise distance were reconstructed for 10,000 iterations with Molecular Evolutionary Genetics Analysis (MEGA X) [32] https://www.megasoftware.net/ and visualized with Tree Figure Drawing Tool (FigTree 1.4.4: http://tree.bio.ed.ac.uk/software/figtree/). Bootstrap values of 100 were shown. Nucleotide sequences for H5N1 and B/Yamagata strains were unavailable (A/India/NIV/06, B/Oklahoma/10/18 and B/Bangladesh/5972/907, respectively).

### 2.9. Statistical Analysis

Data were analyzed using GraphPad Prism 9 (San Diego, CA, USA). Antisera titers were expressed as mean (±standard deviation) from five mice per group. Two-way ANOVA tests were used to compare immune responses elicited by the low-dose and high-dose vaccines, as well as between intramuscular and intradermal routes of administration, using Šidák correction for multiple comparisons unless otherwise stated (significance threshold set at *p* < 0.05).

## 3. Results

### 3.1. Antisera Responses to Composite Peptide Immunogens and Individual Peptide Components

**Figure 1:**
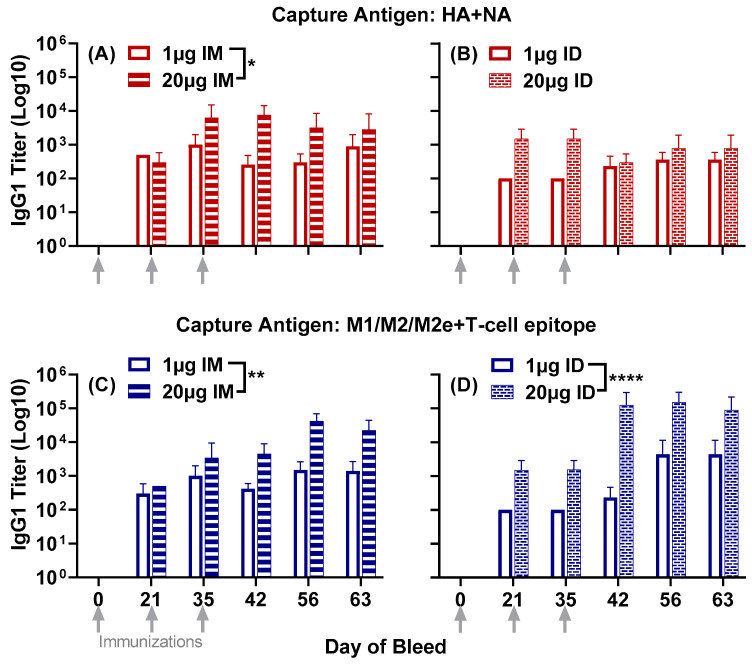
Serum IgG1 responses to the composite peptides HA + NA and M1/M2/M2e + T-cell epitope in mice immunized with either 1 µg or 20 µg of LHNVD-105 formulated with ALFQ, administered intramuscularly or intradermally. Day 0, 21, 35, 42, 56 and 63 sera samples were analyzed using ELISA. IgG1 titers to HA + NA (Flu Pep11) in intramuscular (**A**) and intradermal (**B**) groups; M1/M2/M2e + T-cell epitope (Flu Pep5906) titers in intramuscular (**C**) and in intradermal (**D**). Data are expressed as mean ± SD. * *p* = 0.01, ** *p* = 0.001, **** *p* < 0.0001.

The immunization of mice with LHNVD-105, adjuvanted with ALFQ, generated robust immune responses at both the low dose (1 µg) and the high dose (20 µg) per mouse, administered either intramuscularly or intradermally. There was no observed reactogenicity at the injection site (Supplemental Figure S1). We did not observe any ruffled furs, hunched postures, squinted eyes, mobility issues or eating/drinking issues. Antibody responses in pre-immune sera were undetectable. Serum IgG1 titers to HA + NA (Flu Pep11) and M1/M2/M2e + T-cell epitope (Flu Pep5906) were observed in all groups twenty-one days post primary immunization. Titers to each composite peptide were enhanced after the two boosts at day 21 and day 35 and remained steady up to day 63 (Figure 1).

The high-dose (20 µg) administered either intramuscularly or intradermally generated peak titers to M1/M2/M2e + T-cell epitope (titers up to 10^5^) a log higher than HA + NA (titers up to 10^4^) between day 42 and day 63 (Figure 1). The high-dose intramuscular group generated significantly higher titers to the composite HA + NA peptide than the low-dose (1 µg) intramuscular group (*p* = 0.0143) (Figure 1A).

When comparing the two administration routes, overall titers to HA + NA in the high-dose intramuscular group were significantly higher than the high-dose intradermal group (*p* = 0.0107) (comparison between high-dose groups in Figure 1A,B).

IgG1 titers to the composite peptide M1/M2/M2e + T-cell epitope were significantly enhanced in mice that received the high dose of vaccine, either intramuscularly (*p* = 0.0031) (Figure 1C) or intradermally (*p* < 0.0001) (Figure 1D), compared to the low dose for each route, respectively.

**Figure 2:**
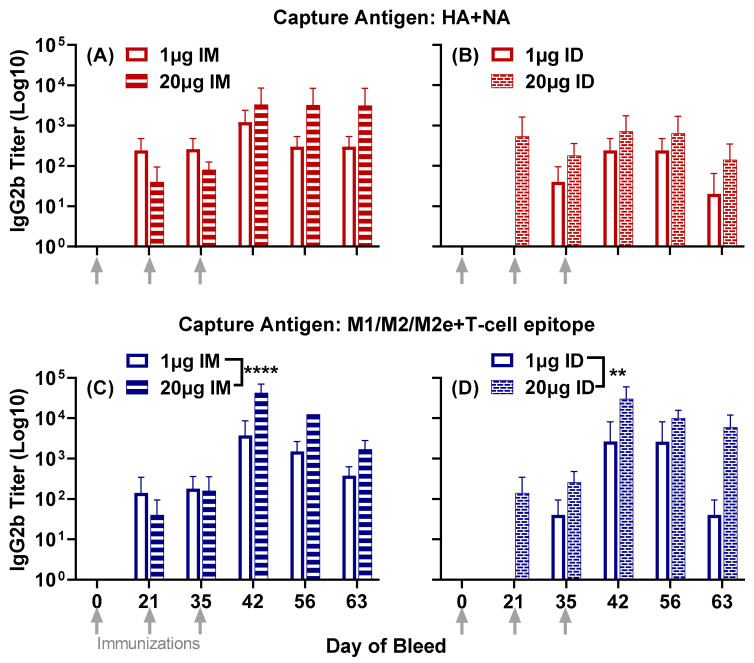
Serum IgG2b responses to the composite peptides HA + NA and M1/M2/M2e + T-cell epitope in mice immunized with either 1 µg or 20 µg of LHNVD-105 formulated with ALFQ, administered intramuscularly or intradermally. Day 0, 21, 35, 42, 56 and 63 sera samples were analyzed using ELISA. IgG2b titers to HA + NA (Flu Pep11) in intramuscular (**A**) and intradermal (**B**) groups; M1/M2/M2e + T-cell epitope (Flu Pep5906) titers in intramuscular (**C**) and intradermal (**D**). Data are expressed as mean ± SD. ** *p* = 0.001, **** *p* < 0.0001.

IgG2b titers to HA + NA and M1/M2/M2e + T-cell epitope were observed 21 days post primary immunization in three out of the four groups: low- and high-dose intramuscular groups and the high-dose intradermal group (Figure 2). The low-dose intradermal group generated IgG2b titers at day 35, following the first boost on day 21. Titers to both composite peptides in all four groups were enhanced at day 42, after the second boost at day 35, and remained steady, albeit with a slight reduction at day 63. (Figure 2).

Unlike IgG1 titers to HA + NA, the comparison between administration routes showed significantly higher IgG2b titers in the low-dose intramuscular group compared to the low-dose intradermal group (*p* = 0.0107) (comparison between low-dose groups in Figure 2A,B).

IgG2b titers to M1/M2/M2e + T-cell epitope showed a profile similar to that obtained with IgG1. The high-dose vaccine demonstrated significantly higher titers than the low-dose vaccine when administered intramuscularly (*p* < 0.0001) (Figure 2C) and intradermally (*p* = 0.0019) (Figure 2D).

Overall, the high-dose vaccine demonstrated significantly enhanced antisera responses compared to the low-dose one. Between the two routes of administration, the intramuscular route generated significantly higher titers to the HA + NA composite peptide compared to the intradermal route. However, titers to the M1/M2/M2e + T-cell epitope composite peptide were largely comparable between the two routes of administration.

**Figure 3:**
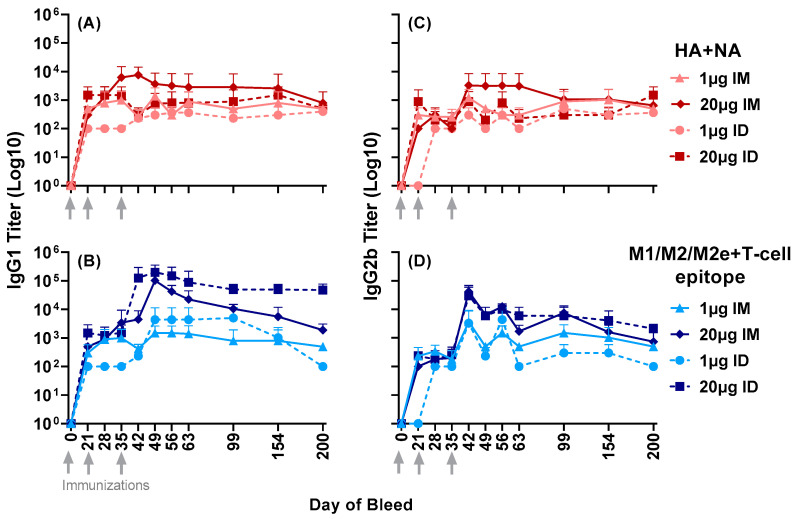
Serum IgG1 and IgG2b responses to the composite peptides HA + NA and M1/M2/M2e + T-cell epitope in mice immunized with either 1 µg or 20 µg of LHNVD-105 with ALFQ, administered intramuscularly or intradermally. Longevity of titers in sera samples up to day 200 was determined using ELISA. (**A**,**C**) demonstrate IgG1 and IgG2b titers to HA + NA (Flu Pep11) and (**B**,**D**), to M1/M2/M2e + T-cell epitope (Flu Pep5906), respectively. Data are expressed as mean ± SD.

Further expanding the antisera profile analysis beyond day 63, both IgG1 and IgG2b titers were observed to HA + NA and M1/M2/M2e + T-cell epitope up to day 200 post primary immunization (Figure 3). This indicated the longevity of antibody responses far beyond the second boost at day 35.

**Figure 4:**
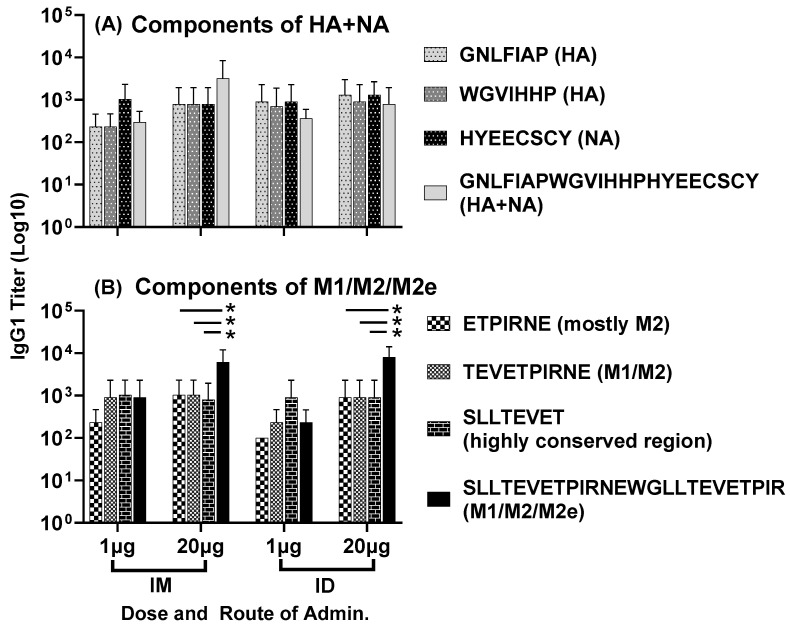
Antisera responses, at day 56, to individual HA, NA and M2e epitopes in mice immunized with LHNVD-105, formulated with ALFQ, administered either intramuscularly or intradermally at two doses of 1 µg of 20 µg. IgG1 titers to individual HA, NA epitopes and the composite peptide HA + NA are shown in panel (**A**) and titers to M1, M2 epitopes and the full-length M1/M2/M2e are shown in panel (**B**). Data from each group (5 mice/group) are expressed as mean ± SD. Statistical significance between titers to individual peptides for each group was calculated using the two-way ANOVA method with Šidák correction for multiple comparison, and the significance threshold set at *p* < 0.05 (* *p* = 0.01). Pre-immunization pooled serum (control) had absorbance values of 0.094–0.104 on different antigens. Endpoint titers are the reciprocal of highest dilution that yielded absorbance values greater than or equal to twice that of the pooled pre-immune values.

IgG1 antisera responses, at day 56, to the highly conserved individual HA and NA epitopes and the composite peptide HA + NA (Flu Pep11) were comparable between the two doses and the two routes of administration (Figure 4A). Titers to the highly conserved individual M1 and M2 epitopes were also comparable between the two doses and routes of administration. However, the high-dose vaccine administered intramuscularly or intradermally generated significantly higher IgG1 titers to the full-length M1/M2/M2e peptide compared to the individual M1 and M2 epitopes (*p* = 0.01) (Figure 4B). Notably, the composite peptide HA + NA was the peptide Flu Pep11 used in the immunogen combination LHNVD-105. The full-length M1/M2/M2e peptide, however, was not the immunogen Flu Pep5906, which is a composite peptide comprising M1/M2/M2e and tetanus T-cell epitope (M1/M2/M2e + T-cell epitope). In addition, these day 56 titers to HA + NA and M1/M2/M2e (without T-cell epitope) (Figure 4A,B) demonstrate that the enhanced titers to M1/M2/M2e + T-cell epitope shown in Figure 1 above (with the high-dose (20 µg) vaccine) was not due to the presence of T cell epitope.

**Figure 5:**
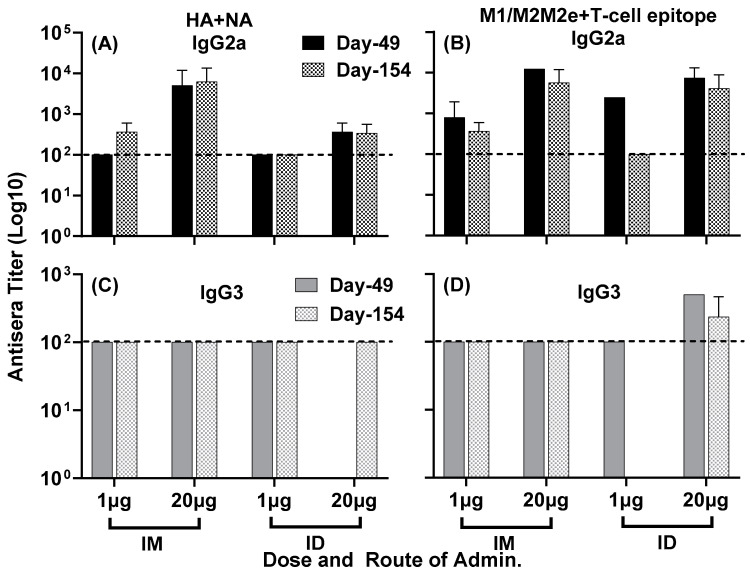
Isotype-specific IgG antisera responses, at day 49 and day 154, in mice immunized with LHNVD-105 with adjuvant ALFQ. IgG2a titers to HA + NA and M1/M2/M2e + T-cell epitope at day 49 and day 154 are shown in panels (**A**,**B**), and IgG3 titers at day 49 and day 154 are demonstrated in panels (**C**,**D**), respectively. Data from each group (5 mice/group) are expressed as mean ± SD. Pre-immunization pooled serum (control) had absorbance values of 0.053–0.076 with different IgG isotypes. Endpoint titers are the reciprocal of highest dilution that yielded absorbance values greater than or equal to twice that of the pooled pre-immune values. Dashed line at 100 represents minimum calculated titer. Titers below 100 are reported as nil.

Further analysis of isotype-specific IgG responses showed IgG2a and IgG3 titers to both composite peptides HA + NA (Figure 5A,C) and M1/M2/M2e + T-cell epitope (Figure 5B,D) at day 49, which largely remained steady at day 154 post primary immunization, indicating the durability of these responses.

**Figure 6:**
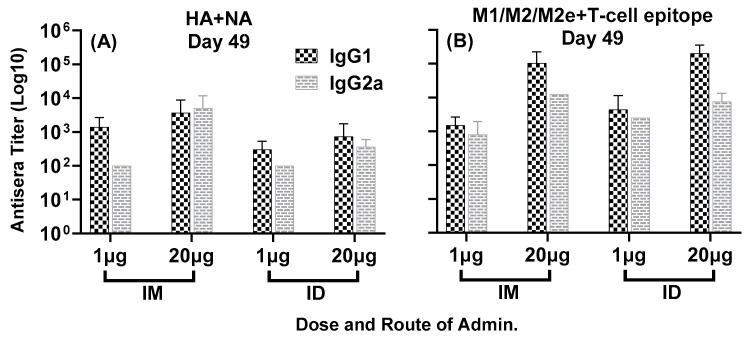
Isotype-specific IgG antisera responses, at day 49, in mice immunized with LHNVD-105 with ALFQ adjuvant. IgG1 and IgG2a titers to HA + NA and M1/M2/M2e + T-cell epitope at day 49 are shown in panels (**A**,**B**), respectively. Data from each group (5 mice/group) are expressed as mean ± SD. Pre-immunization pooled serum (control) had absorbance values of 0.053–0.129 with different IgG isotypes. Endpoint titers are the reciprocal of highest dilution that yielded absorbance values greater than or equal to twice that of the pooled pre-immune values.

Antisera responses generated by the combination of multiple conserved epitopes of HA, NA and M2e, formulated with ALFQ adjuvant, encompassed four different isotypes of IgG: IgG1, IgG2b, IgG2a and IgG3. As demonstrated in Figure 6A,B, all four immunization groups elicited largely comparable IgG1 and IgG2a titers at day 49, representing Th2 and Th1 pathways, respectively. The presence of good Th1 and Th2 responses indicates the engagement of both cellular and humoral immunity by the composite peptide combination vaccine LHNVD-105.

### 3.2. Antisera Titers to Influenza A and B Viruses

**Figure 7:**
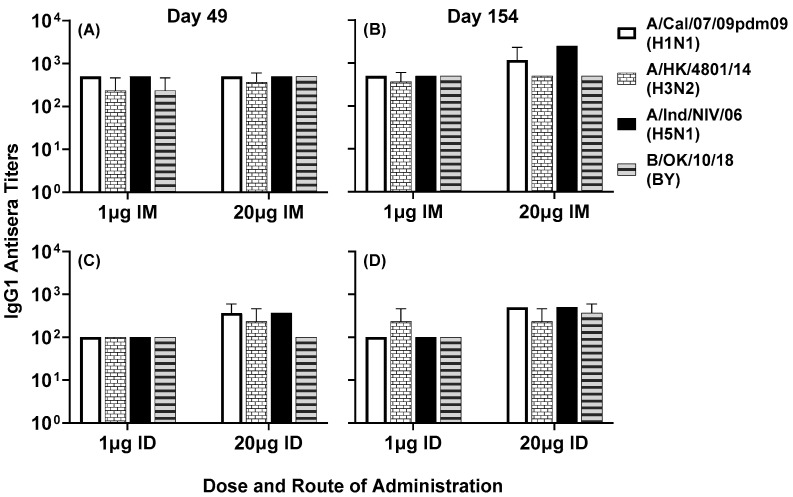
Day 49 (**A**,**C**) and day 154 (**B**,**D**) pooled sera from mice immunized with LHNVD-105 formulated with ALFQ, at two doses of 1 and 20 µg administered intramuscularly or intradermally, respectively, recognized live/inactivated contemporary influenza A virus of subtypes H1N1, H3N2, H5N1 and live influenza B virus of Yamagata lineage. IgG1 titers to viruses in pooled sera from each group were analyzed using ELISA. Data expressed as mean ± SD from three separate experiments with pooled sera from each group. Pre-immunization pooled serum (control) had mean absorbance values of 0.110–0.144 on different virus strains. Endpoint titers are the reciprocal of highest dilution that yielded absorbance values greater than or equal to twice that of the pooled pre-immune values.

Serum antibody responses to various subtypes of influenza A and B viruses were analyzed by ELISA. IgG1 titers to A/Cal/07/09pdm09 (H1N1), A/HK/4801/14 (H3N2), A/Ind/PR8/06 (H5N1) and B/OK/10/18 (BY) were observed in all four groups at day 49 (Figure 7A,C) and day 154 (Figure 7B,D). In the intradermal group, the high-dose vaccine generated slightly higher titers compared to the low-dose vaccine at day 49 (Figure 7C) and day 154 (Figure 7D). In the intramuscular group, low- and high-dose vaccines generated comparable titers at both time points (Figure 7A,B). In the high-dose intramuscular group, titers to some virus subtypes were enhanced between day 49 and day 154.

**Figure 8:**
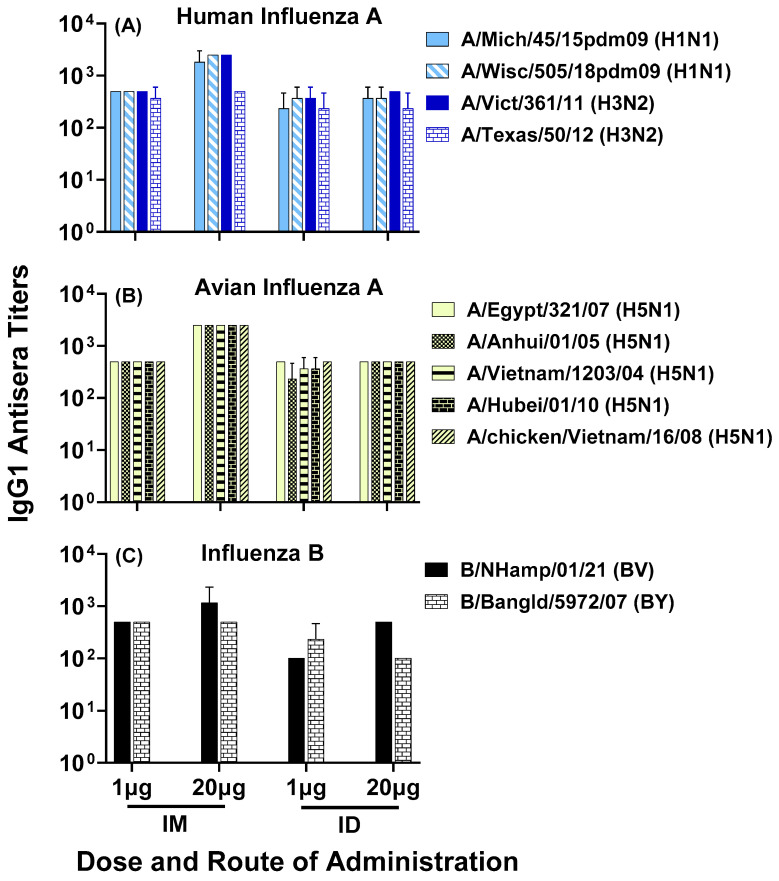
Day 200 pooled sera from mice immunized with LHNVD-105 formulated with ALFQ, at two doses of 1 and 20 µg administered intramuscularly or intradermally, respectively, recognized contemporary live influenza A virus of subtypes H1N1 and H3N2 (**A**), inactivated H5N1 (**B**) and live influenza B virus of Victoria and Yamagata lineages (**C**). IgG1 titers to viruses in pooled sera from each group were analyzed using ELISA. Data expressed as mean ± SD from three separate experiments with pooled sera from each group. Pre-immunization pooled serum (control) had mean absorbance values of 0.074–0.096 on different virus strains. Endpoint titers are the reciprocal of highest dilution that yielded absorbance values greater than or equal to twice that of the pooled pre-immune values. No statistical significance was observed between the groups (*p* > 0.05).

To demonstrate that the composite peptide vaccine LHNVD-105, formulated with ALFQ adjuvant, generated broadly active and durable antibody responses to influenza viruses, we analyzed pooled sera from day 200 bleeds and observed comparable antisera titers in all four groups of low- and high-dose vaccine administered intramuscularly or intradermally to various strains of human influenza A groups 1 and 2 (Figure 8A), avian influenza A (Figure 8B) and influenza B (Figure 8C) viruses.

### 3.3. Serum Neutralizing Titers against Group 1 and 2 Influenza A Viruses

**Figure 9:**
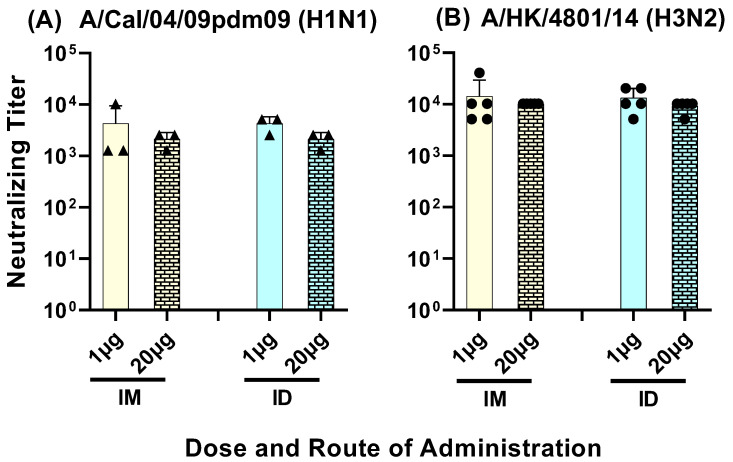
Day 42 to Day 63 serum samples from mice immunized with LHNVD-105 with adjuvant ALFQ neutralized live contemporary influenza A virus of H1N1 (**A**) and H3N2 (**B**) subtypes. Pooled sera from each group were analyzed for virus neutralization using MNA. Titer represents neutralization of the virus in three out of four wells. Negative control titer was at 10^1^. Data from three (triangles in panel A) to five (circles in panel B) separate experiments are expressed as mean ± SD.

Neutralizing antibodies to Group 1 and 2 influenza A viruses were observed in sera from all four groups. The neutralizing titer in pooled sera samples from day 42 and day 63, against a seasonal human A/H3N2 strain, ranged between 5000 and 40,000 (Figure 9A). The neutralizing titer against the 2009 swine fu pandemic A/H1N1pdm09 strain ranged between 1000 and 10,000 (Figure 9B). No significant differences were observed in the neutralizing titers between the low- and high-dose vaccine administered intramuscularly or intradermally.

Phylogenetic analyses demonstrated that this vaccine has the potential to induce binding and neutralizing antibodies against human and avian influenza strains, where neighbor-joining trees demonstrated broad antisera cross-reactivity to globally circulating strains of H1N1, H3N2, H5Nx and B/Victoria influenza viruses (Supplemental Figure S2).

## 4. Discussion

Small synthetic peptide vaccines can be easily synthesized and scaled, overcoming the time-consuming process of egg-based production that leads to antigenic changes during manufacture. Multiple conserved epitopes from HA, NA and Matrix (M1/M2/M2e) with a universal T cell epitope are combined to form composite peptide vaccine antigens that promote broad immunity to group 1 and 2 influenza virus strains [14]. In addition, the selection of a suitable adjuvant to improve the immunogenicity of a small peptide vaccine is one of the most significant aspects of designing a peptide vaccine. The formulation of influenza composite peptides containing highly conserved epitopes from HA head and stalk regions, NA and Matrix protein provides broad and potent stimulation of the immune system at many points while maintaining a balanced Th1 and Th2 response [14].

Another aspect to consider for vaccine efficacy is the route of administration. In the standard intramuscular route, a vaccine is delivered into the muscle tissue and subsequently circulates into the draining lymph nodes to be presented to the resident antigen-presenting cells (APC), which in turn activate the B cells and T cells. Other routes of vaccine delivery are being explored due to lessons learned from the COVID-19 pandemic vaccine supply shortages. One example is the intradermal route of immunization, where a vaccine is delivered into the skin and generates strong and long-lasting immune responses from a plethora of skin-resident APCs such as dermal dendritic cells, Langerhans cells and macrophages [33]. The intradermal route of administration is steadily gaining recognition, with an advantage of being dose-sparing, and with the advent of new tools such as needle-free applications, thereby making it cost-effective for scaling-up and providing a greater degree of responsiveness during public health emergencies or with the challenges posed by influenza outbreaks in poultry and swine populations [34,35,36,37].

Our previous publication showed that an ALFQ-adjuvanted composite influenza peptide vaccine comprising multiple highly conserved epitopes of HA, NA and Matrix (M1/M2/M2e) and delivered intramuscularly at a low dose of 1 µg per mouse generated a strong and balanced immune response with cross-neutralizing antibodies against Group 1 and Group 2 influenza viruses [14]. The ALFQ adjuvant improved the immunogenicity of the peptide vaccine and circumvented the need for conjugation of the small peptide vaccine. In addition, the presence of a tetanus toxoid universal T cell epitope on the composite Matrix (M1/M2/M2e) peptide further enhanced and increased the breadth of immune responses to the peptide vaccine. A balanced immune response including both Th1 and Th2 cytokines was generated, thereby demonstrating the activation of both humoral and cellular branches of immunity.

In this study, we demonstrate that two doses, 1 µg and 20 µg per mouse, of our multi-epitope composite peptide vaccine formulated with ALFQ adjuvant and administered using two different routes, intramuscular and intradermal, generated strong, balanced, and long-lasting immune responses in mice, with broadly neutralizing antibodies to human A/H3N2 and 2009 swine flu pandemic A/H1N1pdm09 strains. Since variable culture conditions are required for each virus strain, the neutralization assay may need to be optimized for each subtype and emerging influenza virus strains. The antisera recognized several strains of human, swine and avian influenza A and influenza B viruses. The high dose of 20 µg per mouse of the composite influenza peptide vaccine showed significantly higher immune responses to peptide components of the vaccine HA + NA (Flu Pep11) and M1/M2/M2e + T-cell epitope (Flu Pep5906) compared to the 1 µg low dose, given either intramuscularly or intradermally. However, no significant functional differences were observed between the two doses in both binding and neutralizing capacities against various subtypes of influenza A and B viruses up to 200 days post primary immunization, thus indicating that the low-dose (1 µg) vaccine might be sufficient to elicit a robust functional immune response, when administered either through the traditional intramuscular route or the novel intradermal route. Notably, the serum antibodies demonstrated recognition of the zoonotic 2009 swine flu pandemic A/H1N1 strain, the highly pathogenic avian influenza A/H5N1 and the influenza B strains of Victoria and Yamagata lineages. Additionally, strong neutralizing titers were obtained against both seasonal human A/H3N2 and 2009 swine flu pandemic A/H1N1pdm09 influenza strains. Due to non-availability of mouse serum samples, additional microneutralization assays could not be conducted. Phylogenetic analyses demonstrated that this vaccine has the potential to induce binding and neutralizing antibodies against human and avian influenza strains. In addition, others have shown that vaccines that targeted matrix peptides elicited antibodies to H5N1, H1N1 and H7N9 which were protective against lethal challenge with these strains [38,39,40]. These data support the conclusion that this multi-epitope composite peptide influenza vaccine, that includes HA, NA and matrix peptides, will provide broad neutralization and protection across influenza subtypes.

Considering the impact of avian influenza outbreaks of the A/H5N1 subtype, with occasional transmission to humans leading to a threat of another zoonotic pandemic, and the prevalence of influenza A/H1N1, A/H3N2 and influenza B strains in seasonal outbreaks among humans and pigs, this composite peptide vaccine formulated with ALFQ adjuvant provides an important strategy for developing a universal influenza vaccine.

## 5. Conclusions

Our previous publication showed the generation of robust and balanced immune responses to the composite peptide influenza vaccine composed of highly conserved HA, NA and Matrix (M1/M2/M2e) epitopes formulated with ALFQ adjuvant [14]. As an extension of our previous study, we confirm that the low dose (1 µg) of our composite peptide vaccine adjuvanted with ALFQ is adequate in generating robust immunogenicity. A major novelty of the current study lies in the induction of durable antibody responses (up to day 200 post primary immunization) following the administration of the influenza peptide vaccine by the intradermal route as well as the intramuscular route. In addition to the broad cross-reactivity of immune responses to influenza viruses beyond the 2009 swine flu pandemic A/H1N1pdm09 and seasonal human A/H3N2 strains previously reported, we also show the unique cross-reactivity to the highly pathogenic avian influenza A/H5N1 and the influenza B strains of Victoria and Yamagata lineages in response to unconjugated peptides.

Therefore, this study further demonstrates broad and durable immune responses to peptides and multiple subtypes of influenza viruses. Induced durable immunity across human, swine, and avian influenza viruses, including A/H5N1, neutralized both seasonal and pandemic strains. Moreover, comparable immune responses to influenza viruses were observed with intramuscular and intradermal routes of administration, thereby providing a prospect for multiple vaccine delivery options, especially during public health emergencies.

This unconjugated composite peptide vaccine comprising multiple conserved influenza epitopes formulated with a safe and highly potent adjuvant ALFQ, and administered by intramuscular and intradermal routes, offers an alternative strategy to protein-subunit-based or nucleic-acid-based vaccines by providing a cost-effective, easily scalable supra-seasonal vaccine that generates broad and durable immunity to human and avian influenza viruses, thus moving us closer to a universal influenza vaccine and pandemic preparedness.

## Data Availability

The data are available from the corresponding author upon request.

## Supplementary Materials

The following supporting information can be downloaded at: https://www.mdpi.com/article/10.3390/vaccines11091468/s1, Figure S1: Representative images of injection site; Table S1: Influenza virus strains; Figure S2: Phylogenetic analysis.
