# Supplementary material for: Unconjugated Multi-Epitope Peptides Adjuvanted with ALFQ Induce Durable and Broadly Reactive Antibodies to Human and Avian Influenza Viruses
Source: Vaccines (Basel). 2023 Sep 8;11(9):1468. doi: 10.3390/vaccines11091468 (PMC10537791; doi:10.3390/vaccines11091468)
Supplement: Supplementary file 1 [file vaccines-11-01468-s001.zip › vaccines-2510481-supplementary.pdf]

Supplemental Figures:

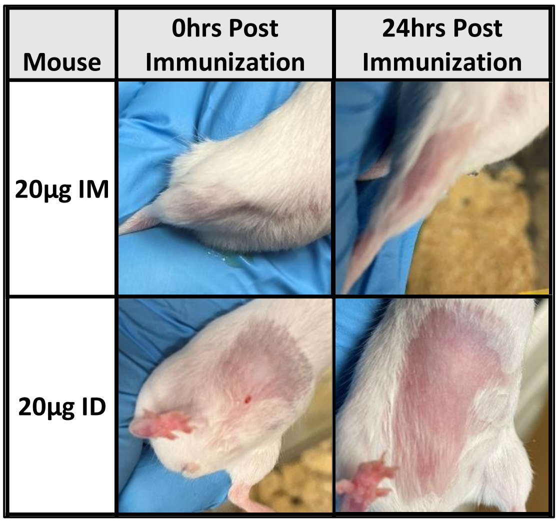

Supplemental Figure S1. Representative images of injection sites in mice immunized with 20 µg of LHNVD-105, administered either intramuscularly or intradermally. Post-immunization images were taken immediately, 24hrs and 72hrs after primary immunization.

Types/Subtypes	Clade	Strains	Annotation
A/H1N1	*	live A/California/07/2009pdm09	A/Cal/07/09pdm09
	**	live A/California/04/2009pdm09	*A/Cal/04/09pdm09
	6B.1	live A/Michigan/45/2015pdm09	A/Mich/45/15pdm09
	6B.1A.5	live A/Wisconsin/505/2018pdm09	*A/Wisc/505/18pdm09
A/H3N2	3C.2a	live A/Hong Kong/4801/2014	A/HK/4801/14
	3C.1	live A/Victoria/361/2011	A/Vict/361/11
	3C.1	live A/Texas/50/2012	A/Texas/50/12
A/H5N1 (avian)	2.3.2.1a	BPL-inactivated A/India/NIV/2006-PR8-IBCDC-RG7	A/Ind/NIV/06
	2.2.1	BPL-inactivated Influenza A/Egypt/321/2007 x PR8-IDCDC-RG11	A/Egypt/321/07
	2.3.4	BPL-inactivated Influenza A/Anhui/01/2005 x PR8-IBCDC-RG6	A/Anhui/01/05
	1	BPL-inactivated Influenza A/Vietnam/1203/2004 x PR8-CDC-RG/GLP	A/Vietnam/1203/04
	2.3.2.1	BPL-inactivated Influenza A/Hubei/1/2010 x PR8-IDCDC-RG30	A/Hubei/01/10
	7.1	BPL-inactivated Influenza A/chicken/Vietnam/NCVD-016/2008 x PR8-IDCDC-RG12	A/chicken/Vietnam/16/08
B/Victoria		live B/New Hampshire/01/2021	B/NHamp/01/21
B/Yamagata		live B/Oklahoma/10/2018	B/OK/10/18
		live B/Bangladesh/5972/2007	B/Bangld/5972/07

* 2010-2016 vaccine strain

**Reference virus

Supplemental Table S1: Influenza virus strains

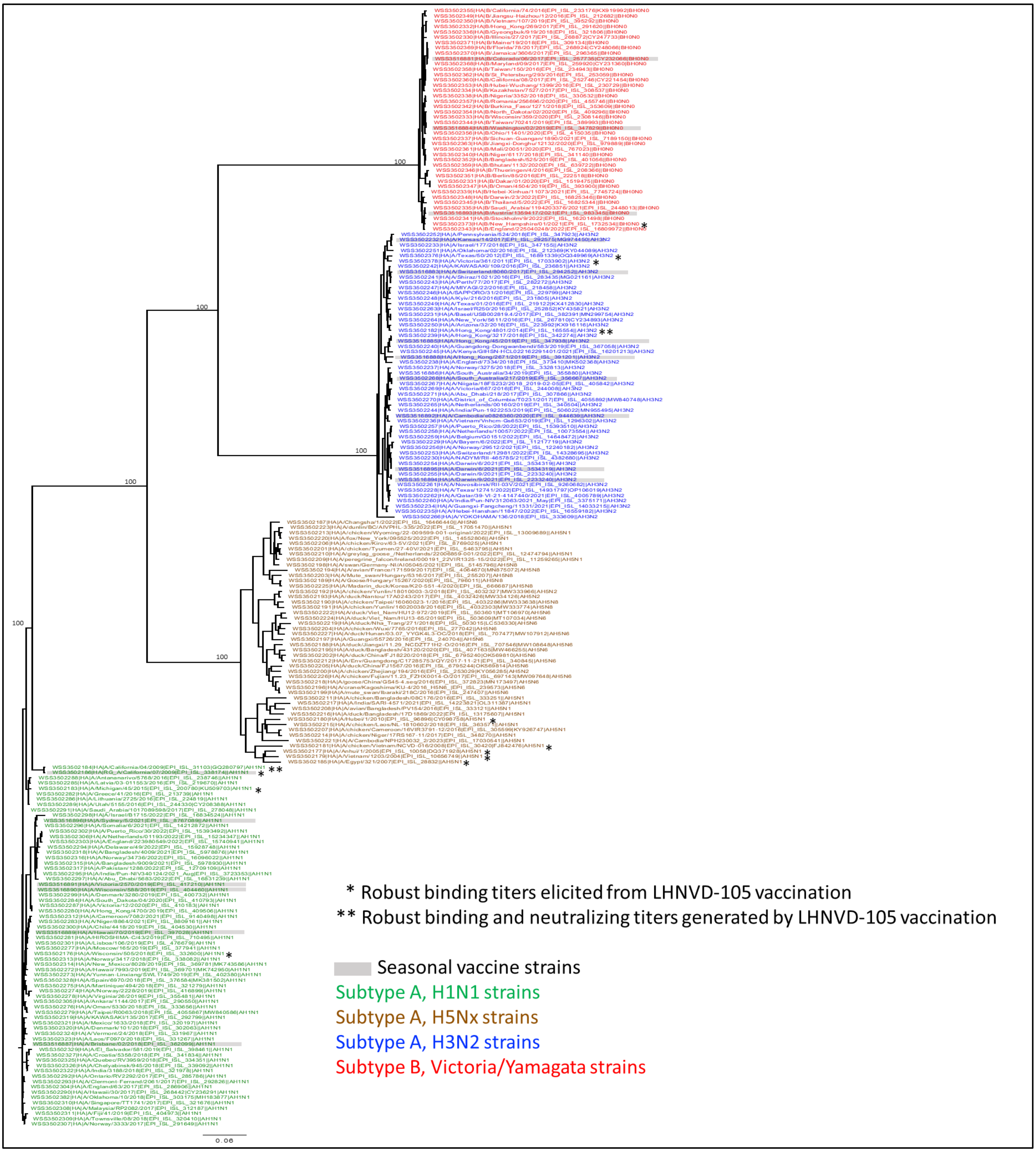

Supplemental Figure S2: Neighbor-joining trees based on pairwise distance of influenza strains for which binding (*) and neutralizing (**) antibody titers were generated *in vivo* after LHNVD-105 immunization. Bootstrap value of 100 are shown. Green = Subtype A H1N1 strains. Brown = Subtype A H5Nx strains. Blue = Subtype A H3N2 strains. Red = Subtype B Victoria/Yamagata strains. Grey = Seasonal vaccine strains. EPI_SET Identifier can be found in taxa.
